# The Critical Role of Oxidative Stress in Sarcopenic Obesity

**DOI:** 10.1155/2021/4493817

**Published:** 2021-10-12

**Authors:** Andrea Gonzalez, Felipe Simon, Oscar Achiardi, Cristian Vilos, Daniel Cabrera, Claudio Cabello-Verrugio

**Affiliations:** ^1^Laboratory of Muscle Pathology, Fragility and Aging, Department of Biological Sciences, Faculty of Life Sciences, Universidad Andres Bello, Santiago 8370146, Chile; ^2^Millennium Institute on Immunology and Immunotherapy, Santiago 8370146, Chile; ^3^Center for the Development of Nanoscience and Nanotechnology (CEDENNA), Universidad de Santiago de Chile, Santiago 8350709, Chile; ^4^Millennium Nucleus of Ion Channel-Associated Diseases (MiNICAD), Universidad de Chile, Santiago 8370146, Chile; ^5^Laboratory of Integrative Physiopathology, Department of Biological Sciences, Faculty of Life Sciences, Universidad Andres Bello, Santiago 8370146, Chile; ^6^Escuela de Kinesiología, Facultad de Ciencias, Pontificia Universidad Católica de Valparaíso, Valparaíso 2340025, Chile; ^7^Laboratory of Nanomedicine and Targeted Delivery, Center for Medical Research, School of Medicine, Universidad de Talca, Talca 3460000, Chile; ^8^Departamento de Gastroenterología, Facultad de Medicina, Pontificia Universidad Católica de Chile, Santiago 8330077, Chile; ^9^Facultad de Ciencias Médicas, Universidad Bernardo O Higgins, Santiago 8370993, Chile

## Abstract

Sarcopenic obesity (SO) is a combination of obesity and sarcopenia that primarily develops in older people. Patients with SO have high fat mass, low muscle mass, low muscle strength, and low physical function. SO relates to metabolic syndrome and an increased risk of morbimortality. The prevalence of SO varies because of lacking consensus criteria regarding its definition and the methodological difficulty in diagnosing sarcopenia and obesity. SO includes systemic alterations such as insulin resistance, increased proinflammatory cytokines, age-associated hormonal changes, and decreased physical activity at pathophysiological levels. Interestingly, these alterations are influenced by oxidative stress, which is a critical factor in altering muscle function and the generation of metabolic dysfunctions. Thus, oxidative stress in SO alters muscle mass, the signaling pathways that control it, satellite cell functions, and mitochondrial and endoplasmic reticulum activities. Considering this background, our objectives in this review are to describe SO as a highly prevalent condition and look at the role of oxidative stress in SO pathophysiology.

## 1. Introduction

Sarcopenic obesity (SO) was described in 1996 by Heber et al. [1], but it is not clearly defined. Nevertheless, the most accepted definition of SO is a combination of obesity and sarcopenia, mainly, although not exclusively, in older people. SO is characterized by high fat mass, low muscle mass, low muscle strength, and low physical functionality [1–7]. People that develop SO are primarily over 60 years old, with comorbidities such as type 2 diabetes mellitus (T2DM), nonalcoholic fatty liver diseases (NAFLD), dyslipidemia, hypertension, and cardiovascular disease. They generally have a sedentary lifestyle and engage in harmful habits such as tobacco and alcohol consumption and a high-fat and/or carbohydrate diet [3].

SO associates with a high risk of hospitalization, loss of independence, disability, frailty, increased risk of fractures, impaired quality of life, higher mortality, and multimorbidity [8–12]. Thus, SO decreases the physical functional capacity to a higher degree than sarcopenia or obesity separately [13, 14]. Considering this background, SO is regarded as a severe public health problem.

SO relates to metabolic syndrome (hypertension, hyperglycemia, insulin resistance (IR), T2DM, abnormal lipid metabolism, and dyslipidemia) and lower cardiorespiratory fitness [11, 15–19]. IR, high proinflammatory cytokine levels, hormonal changes due to aging, decreased physical activity, and oxidative stress (Os) all promote SO and are common in the pathophysiology of obesity and sarcopenia. Among these factors, Os is a critical factor in the development of aging and obesity and, therefore, strongly influences SO. This review is aimed at describing SO as a highly prevalent condition and examining the role of Os in its pathophysiology.

## 2. Sarcopenic Obesity: General Characteristics

The prevalence of SO varies between 2% and 85%. This wide range depends on the heterogeneity of SO definitions, the analyzed population, and the different criteria and/or diagnostic methods of obesity and sarcopenia [7, 19–21].

For SO diagnosis, it is essential to consider sarcopenia and obesity. As such, it is difficult to reach a consensus due to the multiple methods of evaluation for each condition, the use of some imprecise techniques (such as body mass index (BMI)), and the existence of different cut-off points for some values according to the population to be evaluated [12] (see Table 1). Actually, the SO diagnosis achieves through an assessment of skeletal muscle mass measured by computed tomography (CT) at the L3 level corrected for height squared (named skeletal muscle index) and BMI (>25 or 30 kg/m^2^). However, there are no internationally standardized criteria for diagnosing SO [22]. Other diagnostic methods include dual X-ray absorptiometry (DXA), magnetic resonance imaging (MRI), and bioimpedance analysis (BIA). Still, all these methods are complex and costly and less frequently used in clinical practice. These methodologies are also a challenge to perform large-scale research and compare data between studies [19]. For these reasons, it is crucial to identify feasible methods for clinical use that allow a precise diagnosis of SO.

Since SO is composed of sarcopenia and obesity, we shall provide details of the relevant aspects of both conditions.

### 2.1. Sarcopenia

Sarcopenia is defined as a “syndrome with progressive and generalized loss of skeletal muscle mass, strength and physical function, which in turn is associated with an increased risk of adverse outcomes, such as physical disability, poor quality of life and higher mortality” [23–25]. It is classified as primary (associated with aging) or secondary (associated with limited mobility, malnutrition, or chronic diseases, such as obesity) [26, 27]. According to the European Working Group on Sarcopenia in Older People (EWGSOP), the diagnosis of sarcopenia is based on the presence of three criteria: (i) loss of muscle strength (a leading indicator of sarcopenia), (ii) decrease in the quantity or quality of muscle mass, and (iii) low physical performance [23, 26, 28]. The sarcopenia diagnosis is challenging due to the different tests and commonly used tools. In Table 1, we describe the primary diagnostic forms of sarcopenia in both clinical and research settings.

Sarcopenia is clinically relevant because the World Health Organization (WHO) has recognized it as a disease and included it in the International Classification of Diseases (ICD code M62.8) [29]. Furthermore, it is a critical determinant of frailty that leads to loss of autonomy and functionality in daily activities. Besides, sarcopenia increases hospitalization, osteoarthritis, osteoporosis, and the risk of institutionalization [30].

### 2.2. Obesity

The WHO defines obesity as “abnormal or excessive fat accumulation that may impair health” and an obese person as someone with a body mass index (BMI) greater than or equal to 30 [31, 32]. The WHO recognizes obesity as a chronic and progressive disease with a high chance of relapse, so it is considered a world epidemic [33]. The obesity diagnosis can be achieved in clinical settings through BMI, waist circumference, waist-to-hip ratio (WHR), waist-to-height ratio (WHTR), and fat mass. In the research context, obesity is usually diagnosed using DEXA, US, and BIA (see Table 1) [34–36].

The obesity diagnosis is marked by difficulties, particularly in relation to BMI. Although BMI is widely used around the world to diagnose obesity, it is an imprecise method because it does not discern between lean and fat mass, neither does it specify fat quantity or distribution [37]. Also, ethnic differences in BMI values have been observed (e.g., Asian population) [38, 39]. Furthermore, BMI is not the best method to determine obesity in the elderly because there are changes in the body composition during aging (body fat redistribution and muscle mass and bone density reductions), affecting the cut-off points for BMI [8, 34, 40].

Obesity is a risk factor in developing other diseases such as cardiovascular diseases (atherosclerosis, myocardial infarct, heart failure, and coronary disease), metabolic syndrome, T2DM, NAFLD, cirrhosis, cancer, osteoarthritis, pulmonary dysfunction (e.g., obstructive sleep apnea syndrome), reduced cognitive skills, urinary incontinence, and, more recently, coronavirus disease 2019 (COVID-19) [41–48].

## 3. Pathophysiology of Sarcopenic Obesity

**Figure 1:**
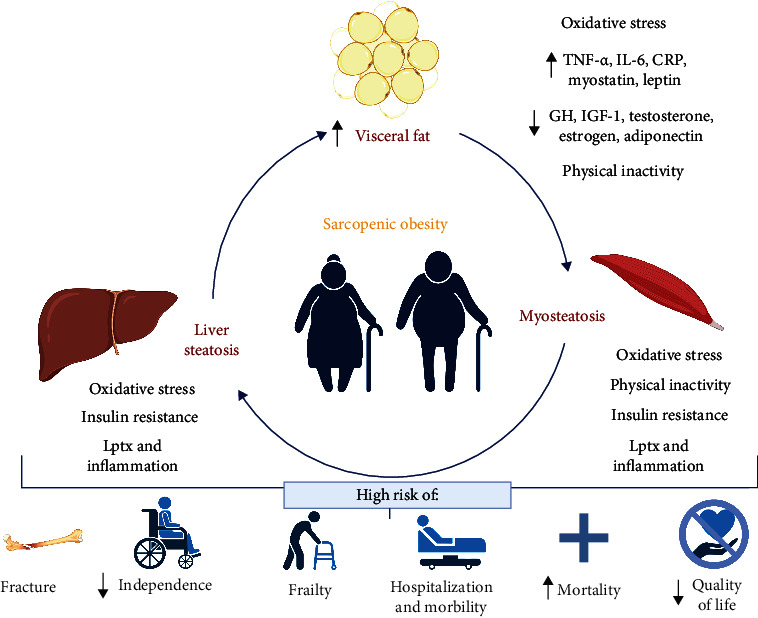
Pathophysiology and consequences of sarcopenic obesity. Sarcopenic obesity (SO) is a combination of obesity and sarcopenia in older people. Obesity and sarcopenia share pathological alterations such as insulin resistance, increased proinflammatory cytokines, age-associated hormonal changes, decreased physical activity, oxidative stress, and liver, adipose, and skeletal muscle dysfunction. Increased body fat mass, especially in the abdominal area (visceral fat), is characteristic of obesity and aging and produces an accumulation of adipose tissue in the liver (liver steatosis) and skeletal muscle (myosteatosis), with the consequent induction of IR, lipotoxicity (Lptx), inflammation, and oxidative stress (Os). Adipocyte hypertrophy induces a state of chronic systemic inflammation characterized by decreased adiponectin and elevated levels of C-reactive protein (CRP), leptin, tumor necrosis factor-*α* (TNF-*α*), and interleukin 6 (IL-6). Also, obesity and aging produce hormonal changes such as a decrease in growth hormone (GH), testosterone, estrogen, IGF-1, and adiponectin and an increase in myostatin. Finally, physical inactivity is a common feature of obesity and aging, affecting respiratory, osteoarticular, and neuromuscular levels, inducing loss of physical function. The consequences of sarcopenic obesity are a high risk of fractures, frailty, hospitalization, morbidity and mortality, loss of independence, and decreased quality of life. Abbreviations: SO: sarcopenic obesity; Lptx: lipotoxicity; Os: oxidative stress; CRP: C-reactive protein; TNF-*α*: tumor necrosis factor-*α*; IL-6: interleukin 6; GH: growth hormone; IGF-1: insulin-like growth factor 1.

Obesity and sarcopenia have common pathological features that could promote their development, such as IR, increased proinflammatory cytokines, age-associated hormonal changes, decreased physical activity, and Os, as well as liver, adipose, and skeletal muscle dysfunction. In this review, we focus on establishing how these factors affect skeletal muscle to generate sarcopenia. We also emphasize the role of Os in the pathophysiology of SO (Figure 1).

### 3.1. Insulin Resistance

IR is a feature of aging and obesity in humans and rodents. Aging could increase body fat mass, mainly in the abdominal area (visceral fat), which is most common in women than in men—this is called abdominal obesity. Furthermore, in aging, increased intramuscular (myosteatosis) and intrahepatic (liver steatosis) fat deposits induce IR [31, 32]. Interestingly, the decrease in elevated insulin levels and reduction in fat percentage could reverse obesity in older people [19, 31, 33].

Pathological myosteatosis in aging and obesity is associated with decreased insulin sensitivity and muscle mass and strength loss. The mechanism involves the impaired insulin signaling by interacting with lipidic intermediates such as diacylglycerol (DAG), long-chain acyl-coenzyme A, and ceramide. These interactions at various levels inhibit the GLUT-4 translocation to the sarcolemma. Together with these events, the decreased insulin secretion by the pancreas is derived from elevated concentrations of fatty acids, which induces *β*-cell apoptosis and reduces proliferation of pancreatic cells [14, 16, 34–36].

### 3.2. Inflammatory State: Chronic Systemic Inflammation

Systemic chronic inflammation is the primary factor influencing SO pathophysiology. Thus, the chronic inflammatory state in obesity and aging has harmful effects on skeletal muscle, inhibiting protein synthesis, decreasing oxidative capacity, and developing IR.

In obesity, the activation of macrophages, inflammatory T lymphocytes, and mast cells results from higher fat mass and adipocyte hypertrophy, creating a low proinflammatory state and an imbalance of adipokines. The characteristic profile of soluble factors in obesity and aging, such as decreased adiponectin, elevated levels of C-reactive protein (CRP), leptin, tumor necrosis factor-*α* (TNF-*α*), and interleukin 6 (IL-6), could lead to progressive loss of muscle mass and an increase in fat mass [14, 16, 37–40].

CRP is a marker of systemic inflammation. It increases in the elderly and is related to sarcopenia and SO [41, 42]. High leptin levels in aging and obesity upregulate the proinflammatory cytokines IL-6 and TNF-*α*, reducing insulin-like growth factor 1 (IGF1) activity and decreasing their anabolic actions on skeletal muscle [14, 43, 44]. TNF-*α* is a proinflammatory cytokine that increases in aging and obesity, promotes protein degradation, decreases protein synthesis, and inhibits myogenic differentiation [14, 45]. Also, adiponectin and growth hormone (GH) decrease their secretion in obesity and aging, inducing adverse effects on muscle protein synthesis. This effect could be associated with higher levels of “geriatric cytokines,” such as IL-6 and CRP, which decrease muscle mass and strength [14, 38]. IL-6 is a myokine associated with sarcopenia and is upregulated in older persons [31, 38, 41, 44, 46–48]. Furthermore, aging-induced myosteatosis promotes lipotoxicity (Lptx) and contributes to inflammation [49, 50].

### 3.3. Hormonal Changes

Aging comes with is a decrease in anabolic hormones such as testosterone and GH. In males, the testosterone level (including its precursor dehydroepiandrosterone sulfate) declines in aging up to 1% per year from 30. In women, testosterone also rapidly decreases from 20 to 45 years old. This effect could harm muscle protein synthesis. In obese people, testosterone levels are low [31, 41, 51–53].

GH circulant levels also decrease after 30 years of age at a rate of ∼1% per year. These conditions induce loss of muscle mass and accumulation of visceral fat in the elderly [31, 54–58]. Significantly, high levels of circulating free fatty acids (FFA) in elderly obesity inhibit GH production and decrease plasma levels of IGF-I, associated with low muscle mass.

In menopausal women, low estrogen levels promote muscle mass decrease and fat mass increase, mainly in the abdominal area. The fat mass percentage increases waist circumference and cardiovascular risk. These effects could be mitigated with hormone replacement therapy [37, 59, 60].

Myostatin expression increases in skeletal muscle due to obesity and IR. Thus, it could favor the loss of skeletal muscle in SO [14, 61, 62].

### 3.4. Decrease of Physical Activity

The increase in adipose tissue in obesity can interfere with physical activity, leading to lower energy expenditure, favoring an increase in adipose tissue, and producing a vicious circle. Pathophysiological changes in the respiratory system, such as reduced lung and chest wall compliance caused by excess visceral fat, diminish the expiratory reserve volume (ERV) and functional residual capacity (FRC), increase pleural pressure, and cause ventilation and perfusion (V/Q) imbalance [63–65].

As mentioned earlier, physical inactivity and obesity increase the level of lipid circulation and myosteatosis in skeletal muscle, contributing to a decrease in muscle mass and strength and favoring sarcopenia and physical disabilities [14, 22, 66].

Furthermore, obesity in the elderly can favor joint dysfunction, chronic pain, disabilities relating to activities of daily living, and frailty, damaging functional status more than obesity or sarcopenia alone [31, 67–70].

Regarding aging, the limitation of physical activity can occur due to musculoskeletal disorders associated with advanced age, such as joint pain and stiffness. Sarcopenia can also induce the loss of physical function, leading to decreased physical activity and, therefore, an increase in adipose tissue and an augmented risk of obesity [19, 37]. As mentioned above, myosteatosis has been associated with aging, limiting functional activities due to decreased muscle mass and strength [71]. Muscle fibrosis is another pathological condition in aging. It is characterized by replacing skeletal muscle with fibrous connective tissue and impaired regenerative muscle capacity, decreasing muscle mass and functionality [71, 72].

Also, in aging, there are decreased rest metabolic rates (4% per decade after the age of 50 years), reduced motor neurons, and skeletal muscle metabolic adaptations, which could also favor obesity and loss of muscle mass [14, 31, 37, 73, 74].

### 3.5. Oxidative Stress

Oxidative stress (Os) is an imbalance of oxidant species and antioxidant systems towards an oxidative status, which is characterized by the accumulation of reactive oxygen species (ROS), reactive nitrogen species (RNS), and cellular damage [75–77]. There are endogenous and exogenous sources of ROS and RNS. Endogenous sources include nicotinamide adenine dinucleotide phosphate (NADPH) oxidase, myeloperoxidase (MPO), lipoxygenase, mitochondria, and xanthine oxidase. In contrast, exogenous sources include air and water pollution, tobacco, alcohol, heavy metals, drugs, industrial solvents, cooking pollutants, and radiation [75, 78, 79].

Antioxidants are the defense system against ROS-induced toxicity. Endogenous antioxidants include enzymatic, such as superoxide dismutase (SOD), catalase (CAT), and glutathione peroxidase (GSH-Px), and nonenzymatic, such as bilirubin and *β*-carotene. Exogenous antioxidants include ascorbic acid (vitamin C), *α*-tocopherol (vitamin E), and phenolic antioxidants (such as resveratrol, phenolic acids, flavonoids, selenium, zinc, and acetylcysteine) [75, 80, 81].

Under normal conditions, ROS and RNS play a vital role in metabolism, immune response, and cellular proliferation and differentiation. In pathological conditions, there is increased production of ROS and RNS, together with insufficient antioxidant capacity. Os develops under these conditions, causing damage in organelles, carbohydrates, proteins, nucleic acids, and lipids, favoring their dysfunction [76–78].

## 4. Oxidative Stress in Sarcopenic Obesity

The oxidation-inflammatory theory of aging or “oxi-inflamm-aging” proposes that, during aging, chronic Os impairs the immune system, induces an inflammatory state, and creates a vicious circle of Os-inflammation-Os that damages structures, tissues, and organs [75, 82]. In obesity, high Os is associated with Lptx, inflammation, and IR in the liver, skeletal muscle, and adipose tissue [40, 83].

**Figure 2:**
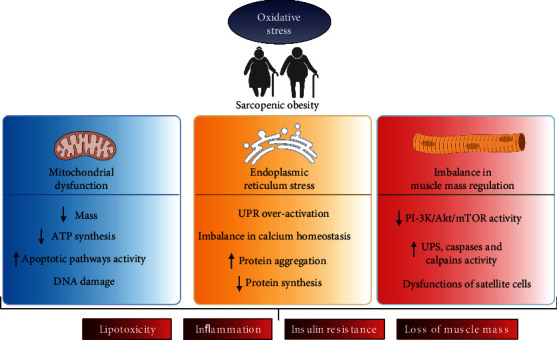
Oxidative stress in sarcopenic obesity. In older people, oxidative stress (Os) favors sarcopenia and obesity through mitochondrial dysfunction, endoplasmic reticulum (ER) stress, and imbalance in muscle mass control. *Mitochondrial dysfunction* in sarcopenia is induced by Os due to mitochondrial DNA damage and impaired mechanisms for repairing DNA ability, impaired capacity to remove dysfunctional mitochondria, decreased mitochondrial quantity and quality, and impaired capacity to generate ATP to activate the apoptotic pathways. *ER stress* and Os are caused by an increase in adipose tissue, chronic inflammation, and insulin resistance, all of which are characteristics of obesity and aging. ER stress induces Os, favoring unfolded protein response (UPR) overactivation, imbalance in calcium homeostasis, increased protein aggregation, and decreased protein synthesis. *Imbalance in muscle mass control* occurs because Os increases the catabolic activity and decreases the anabolic pathway in muscle mass control. Os reduces protein synthesis due to the reduced activity in phosphatidylinositol 3-kinase (PI3K)/serine-threonine kinase (Akt)/mammalian target of rapamycin (mTOR). Os increases the activity of the ubiquitin-proteasome system (UPS) and activates muscle proteases such as caspases and calpains. Finally, due to Os, satellite cells' quantity and regenerative function decline with age and obesity. Mitochondrial dysfunction, ER stress, and imbalance in the muscle mass control pathways induce lipotoxicity (Lptx), chronic inflammation, IR, and loss of muscle mass, affecting physical function and independence in sarcopenic obesity. Abbreviations: Os: oxidative stress; ER: endoplasmic reticulum; UPR: unfolded protein response; PI3K: phosphatidylinositol 3-kinase; Akt: serine-threonine kinase; mTOR: mammalian target of rapamycin; UPS: ubiquitin-proteasome system (UPS); Lptx: lipotoxicity.

Sarcopenia and obesity are associated with Os through mitochondrial dysfunction, endoplasmic reticulum (ER) stress, and imbalance of the muscle mass control pathways. These alterations are detailed below (Figure 2).

### 4.1. Mitochondrial Dysfunction

Os in sarcopenia induces mitochondrial dysfunction due to mitochondrial deoxyribonucleic acid (DNA) damage and impaired mechanisms for repairing DNA by excessive ROS. Moreover, muscle abilities for removing dysfunctional mitochondria become deficient, perpetuating Os [75, 84, 85]. Consequently, there is a decrease in mitochondrial quantity and quality, impairing the capacity to generate adenosine triphosphate (ATP), activating apoptotic pathways, and inducing the loss of muscle fibers [45, 75, 84, 86]. In this regard, aging causes the loss of type II muscle fibers more than type I, probably because type II fibers have a low mitochondrial quantity, making them more susceptible to degradation and loss of muscle mass [75, 87, 88].

In obesity, Os also inhibits mitochondrial function, resulting in Lptx, which impairs insulin signaling (a powerful anabolic signal), promotes high catabolism (which induces muscle mass loss), and leads to IR and inflammation [40, 89].

### 4.2. Endoplasmic Reticulum (ER) Stress

ER stress is induced by ROS accumulation, promoting Os. Obesity, metabolic syndrome, and aging cause ER stress and Os [90–93]. The ER stress and Os in these conditions are related to the “nutrient-sensing” functions of ER, which affect metabolic response at the endocrine and systemic levels [91]. Excess nutrients (ingesting high fat and/or high glucose), chronic inflammatory state, high adiposity, IR, metabolic syndrome, and aging harm the ER function in the liver, skeletal muscle, and adipose tissue, inducing Os [83, 91–95].

In the early stages of metabolic dysregulation, insulin secretion is elevated to compensate for increased glycemia (hyperinsulinemia). The high amount of insulin produced by the pancreas requires that the ER guarantee the correct folding of the hormone, which generates an ER overload and dysfunction, an unfolded protein response (UPR) overactivation, Os, and inflammation [83, 91–95]. Also, with aging and obesity (especially abdominal obesity), insulin sensitivity gradually decreases in skeletal muscle and adipocytes, increasing serum glucose levels and promoting ER overload and Os [83, 91–95].

In the liver, the imbalance in insulin metabolism negatively affects protein synthesis, lipogenesis, lipid transportation, and gluconeogenesis, inducing ER dysfunction and, consequently, Os. Also, adipocytes from obese and insulin-resistant subjects (humans and mice) present elevated lipid storage, lipogenesis, and adipokine synthesis, all of which induce ER stress and Os [83, 91, 96].

ER stress and Os develop during aging due to protein aggregation, damaged or misfolded proteins, an impaired protein cleansing system (by declining autophagic and proteasomal degradation), imbalance in calcium homeostasis, and decreased global protein synthesis. These conditions contribute to decreased skeletal muscle mass in aging [93, 97–101]. Furthermore, physical inactivity, a feature typically observed in aging and obesity, favors ER stress and UPR overactivation, inducing Os [93, 102].

### 4.3. Imbalance in Muscle Mass Control

Skeletal muscles are damaged by Os caused by ROS/RNS accumulation (mainly superoxide anions and hydrogen or peroxyl radicals) and a decrease in antioxidant activity (lower activities of SOD and CAT enzymes). Os leads to an imbalance in protein metabolism, favoring the catabolic pathway and decreasing the anabolic pathway activity. Thus, Os could play a fundamental role in losing the muscle mass that characterizes SO and promote IR [76, 92, 103]. Next, we examine the effects of Os in the control pathways of muscle mass and its impact on satellite cells.

#### 4.3.1. Anabolic Pathway

A critical anabolic way for protein is the phosphatidylinositol 3-kinase (PI3K)/protein kinase B (Akt)/mammalian target of rapamycin (mTOR) pathway. This pathway is stimulated by insulin, insulin-like growth factor 1 (IGF-1), exercise, and testosterone, all of which decrease with obesity and aging [104–106]. Protein synthesis is reduced under Os conditions [107, 108], and Os promotes the activation of pathways such as c-Jun N-terminal kinase (JNK), I*κ*B kinase (IKK), and p38 mitogen-activated protein kinase (p38-MAPK), leading to the inactivation of the insulin receptor. Indeed, they favor IR and low anabolic activity in skeletal muscle [40, 109]. In sarcopenia, the loss of muscle mass and strength leads to reductions in physical activity and mobility, inducing Os, exacerbating sarcopenia, and generating an endless circle [107, 108, 110].

Moreover, in physiological conditions, PI-3K/Akt inhibits the forkhead box transcription factor O (Fox-O) (a potent inductor of the ubiquitin-proteasome system (UPS)), while mTOR decreases caspase activity. PI-3K/Akt/mTOR activity declines during aging, promoting the catabolic pathway. Also, physical inactivity (a characteristic of obesity and sarcopenia in aging) indirectly inhibits the mTOR pathway through Fox-O stimulation [107, 108].

#### 4.3.2. Catabolic Pathway

Elevated ROS levels activate the UPS, increasing expression of the muscle-specific ubiquitin ligase MuRF1 (Muscle RING-finger protein-1) and atrogin-1. ROS also activates muscle proteases such as caspases and calpains, leading to protein breakdown [87, 105, 108, 111, 112].

Another redox-sensitive transcription factor is nuclear factor kappa B (NF-*κ*B), which dramatically increases muscle activity in sarcopenia, metabolic syndrome, and obesity [83, 113]. NF-*κ*B regulates the expression of myokines such as TNF-*α* and IL-6, inducing chronic low-grade inflammation and apoptosis. These conditions increase catabolic pathway activity and decrease anabolic pathway activity in skeletal muscle, promoting the loss of muscle mass and strength [76, 114]. Furthermore, in obesity, NF-*κ*B can be activated by different stimuli such as lipopolysaccharide, free fatty acids (FFAs), advanced glycation end products, inflammatory cytokines, Os, and ER stress. NF-*κ*B activation induces inflammation and IR, favoring catabolic activity, and decreases anabolic pathways in skeletal muscle [76, 83].

#### 4.3.3. Satellite Cells

The function of satellite cells in muscle regeneration and its decline with age contributes to lower capacities to self-renew and regenerate muscle tissue [87, 115, 116]. The reduction in these cells has been explicitly shown in type II fibers more than in type I during aging [87, 117]. Also, myosteatosis, typically displayed in aging, could impair muscle fiber, replace muscle tissue, decrease muscle protein synthesis, and impair new muscle tissue growth [48, 66, 118–120].

The increased ROS levels and decreased antioxidant activity in satellite cells [121, 122] could dysregulate basal autophagy (essential to maintaining the quiescent state of stem cells) and impair the removal of misfolded proteins, thereby affecting satellite cell homeostasis [87, 116]. In addition, Os present in the elderly dysregulates the typical functions and processes of satellite cells such as proliferation, fibrosis, and differentiation involving Notch, Wnt, p38/MAPK, and the JAK-STAT3 signaling pathways [72, 123–125].

Furthermore, satellite cells in obesity and overweight have a minor expression and activity of myogenic regulatory factors (MRFs) such as MyoD, Myf5, and Myf6. MRFs are regulators of the myogenic differentiation of satellite cells in various stages. The decreased activity of MRF is due to dysregulated autophagy concerning inflammation and IR, also leading to a reduction in satellite cells [92, 116, 126, 127].

In obesity, satellite cells acquire adipocyte features, expressing adipocyte-specific genes and accumulating lipids, with a likely effect that favors myosteatosis [128–130].

## 5. Redox-Dependent Mechanisms in Sarcopenic Obesity

There is limited evidence to clarify the mechanisms involved in the redox-dependent effect of SO in human and animal models. Below, we will present information related to the more probable mechanisms involved in Os effects in SO.

### 5.1. Animal Models

Most of the research that links Os and SO in animals is focused on testing agents with antiobesity, antiaging, or antioxidant effects. The results did not directly elucidate the mechanisms by which Os favors SO, but they help understand the associated events to this pathological condition.

Resveratrol, a natural and botanical polyphenol, administered to rats fed with a high-fat diet (HFD), prevented typical SO features such as muscle mass loss, myofiber size decrease, decreased muscle strength, and excessive muscle fat accumulation. The preventive mechanism involved the PKA/LKB1/AMPK pathway [131]. Tocotrienols (TT) and green tea polyphenols (GTP) are other antioxidant agents that increased muscle mass and cross-sectional area (CSA) and increased the mitochondrial enzyme activity in animal models of obesity [132].

Another therapeutical strategy has been the administration of probiotic *Lactobacillus paracasei* PS23 (LPPS23) to aged mice. The effect showed a deacceleration and attenuation of the decline in muscle mass and strength. Mechanistically, treatment with LPPS23 produced a higher mitochondrial function, antioxidant enzymes, and lower inflammatory cytokines and Os [133].

BAM15, a mitochondrially targeted protonophore with wide tolerability, stimulated energy expenditure and glucose and lipid metabolism to prevent diet-induced obesity in mice. Besides, BAM15 improved glycemic control and reduced adiposity through insulin signaling and oxidation of glucose and fatty acids in an AMPK-dependent manner [134].

BDA-410 is a synthetic calpain inhibitor that induced loss of weight and body fat mass in aged mice [135]. In skeletal muscle, BDA-410 improved the skeletal muscle contractility by mechanisms dependent on enhanced lipolysis and excitation-contraction coupling, favoring a lean phenotype [135].

Fucoxanthinol (FXOH) (a metabolite of fucoxanthin (FX) that has antiobesity effects and that accumulates in white adipocytes of mice) showed antisarcopenic and antiobesity activities *in vitro*, mainly by decreasing muscle atrophy, incrementing lipolysis, and decreasing triglyceride (TG) content. Interestingly, the effects of FXOH were dependent on Os [136].

Angiotensin 1-7 (Ang-(1-7)) is a small endogenous peptide that belongs to the renin-angiotensin system [137]. The administration of Ang-(1-7) to mice with obesity or metabolic syndrome reduced body weight, upregulated thermogenesis and brown adipose tissue (BAT), and ameliorated impaired glucose [138]. In obese rats, Ang-(1–7) enhanced glucose tolerance, insulin sensitivity, and decreased plasma insulin levels, as well as a significant decrease in circulating lipid levels [139]. In obese humans, Ang-(1-7) administration decreased vascular dysfunction related to impaired insulin sensitivity [140]. Regarding skeletal muscle, there is broad evidence about the antiatrophic and antisarcopenic role of Ang-(1-7) [141–144]. Despite the fact that the effect of Ang-(1-7) has not been directly assayed in SO, the mechanisms involved in muscle mass regulation include decreased protein degradation, prevention of Os, apoptosis, and mitochondrial dysfunction. These features are separately present in skeletal muscle from obese and aged mice [145–147].

### 5.2. Patients

There is little evidence of the Os-dependent effect in SO patients that could guide the mechanisms involved. Circulating markers of Os (such as GSH, oxidized glutathione (GSSG), plasma malondialdehyde (MDA), and 4-hydroxy 2-nonenal (4-HNE)) increase in elderly patients with SO compared to nonsarcopenic nonobese, sarcopenic nonobese, and nonsarcopenic obese patients. These findings confirm that Os is related to SO [148]. Also, systemic Os is associated with a decline in muscle mass in elderly patients with obesity and T2DM [149]. Furthermore, a study identified biomarkers of inflammation and Os, such as serum adiponectin, the erythrocyte sedimentation rate (ESR), and CRP levels, as being associated with sarcopenia [150]. Interestingly, a recent study showed that a moderate-intensity exercise program reduces oxidative damage and increases the antioxidant system, thereby serving as a feasible tool for treating SO [151].

## 6. Perspectives in Sarcopenic Obesity

Research on the effects of Os on SO is undoubtedly necessary to understand the influence and mechanisms involved in controlling metabolism and muscle mass. It is also essential to harmonize the criteria that define and diagnose sarcopenia and obesity. Similarly, there is a need for advances in reaching a consensus in the methodology for the SO diagnosis that can be applicable in different populations, ideally used in clinical practice, and feasible for use in long-standing studies [3, 7, 12, 16, 37, 48, 152, 153]. Further, since SO is a multifactorial disease, the treatment must also be multifactorial [31]. The treatment could include exercise training and nutritional, pharmacological, psychological, and social interventions [5, 6, 12, 48, 154–156]. One problem is the elevated cost of a multifactorial intervention, which health insurance generally does not cover. Considering these antecedents, prevention could be fundamental. Ideally, the prevention of SO should start early in life, continuing in later stages [31].

## 7. Conclusions

SO is a highly prevalent condition that includes obesity and sarcopenia in aging, which are also highly prevalent. SO increases the risk of physical functional decline in older adults, favoring high morbimortality in patients. The SO diagnosis is the primary difficulty to overcome. There is no consensus on evaluation methods and definitions of SO. Therefore, results from different investigations are highly variable and, thus, difficult to compare.

The pathophysiological factors influencing SO are Os, IR, chronic low-grade inflammation, age-associated hormonal changes, and decreased physical activity. Os is a condition that affects the three main organs and tissues involved in SO (the liver, adipose tissue, and skeletal muscle), leading to a vicious cycle of oxidative damage and inflammation that induces tissue dysfunction. Os-dependent damage due to SO includes mitochondrial dysfunctions and ER stress, which affect the liver, adipose tissue, and skeletal muscle. Also, there is an imbalance in the control muscle mass pathway and satellite cell function that directly affects muscle mass.

## Figures and Tables

**Table 1:** Diagnosis of sarcopenia and obesity.

*Sarcopenia diagnosis*	

Muscle mass	

Clinical setting	Extremity circumferences (thigh, arm) Anthropometry Total or partial body potassium per fat-free soft tissue MAMA (middle-arm muscle area)
Research setting	DEXA (dual-energy X-ray absorptiometry) Thigh US (ultrasound) BIA (bioelectrical impedance analysis) Magnetic resonance imaging (MRI)

Muscle strength	

Clinical setting	Handgrip strength Knee flexion/extension 1 maximum repetition (1RM) 10 maximum repetitions (10RM) Peak expiratory flow (specific to respiratory)
Research setting	Isokinetic evaluation Dynamometer

Physical performance	

Clinical setting	Gait speed Short physical performance battery 6-MWT (6-minute walk test) 2-MST (2-minute step test) Chair stands Timed get-up-and-go test Stair climb power test
Research setting	CPET (cardiopulmonary exercise testing)

*Obesity diagnosis*	

Clinical setting	Body mass index (BMI) (≥30 kg/m^2^)
	Fat mass (FM) % (>25% for men and >35% for women)
	Waist circumference (≥88 cm for women and 102 cm for men)
	Waist-to-hip ratio (WHR)
	Waist-to-height ratio (WHTR)
	Extremity circumferences (thigh, arm)
Research setting	DEXA (dual-energy X-ray absorptiometry)
	US (ultrasound)
	BIA (bioelectrical impedance analysis)

[26, 28, 40, 148, 157–159].

## Abbreviations

Akt: Protein kinase B
AMPK: AMP-activated protein kinase
ATP: Adenosine triphosphate
BAM15: ((2-Fluorophenyl) {6-[(2-fluorophenyl) amino] (1,2,5-oxadiazolo[3,4-e] pyrazin-5-yl)} amine)
BIA: Bioimpedance analysis
BMI: Body mass index
CAT: Catalase
CRP: C-reactive protein
CT: Computed tomography
DAG: Diacylglycerol
DNA: Deoxyribonucleic acid
DXA: Dual X-ray absorptiometry
ER: Endoplasmic reticulum
ERV: Expiratory reserve volume
ESR: Erythrocyte sedimentation rate
EWGSOP: European Working Group on Sarcopenia in Older People
FFAs: Free fatty acids
FRC: Functional residual capacity
FX: Fucoxanthin
4-HNE: 4-Hydroxy 2-nonenal
Fox-O: Forkhead box transcription factor O
FXOH: Fucoxanthinol
GH: Growth hormone
GSH: Reduced glutathione
GSH-Px: Glutathione peroxidase
GSSG: Oxidized glutathione
HFD: High-fat diet
IGF-1: Insulin-like growth factor 1
IKK: I*κ*B kinase
IL-6: Interleukin 6
IR: Insulin resistance
JAK-STAT3: Janus kinase-signal transducer and activator of transcription proteins
JNK: c-Jun N-terminal kinase
LPPS23: Probiotic Lactobacillus paracasei PS23
LKB1: Liver kinase B1
Lptx: Lipotoxicity
MDA: Malondialdehyde
MFRs: Myogenic regulatory factors
MPO: Myeloperoxidase
MRI: Magnetic resonance imaging
mTOR: Mammalian target of rapamycin
MuRF1: Muscle RING-finger protein-1
NADPH: Nicotinamide adenine dinucleotide phosphate
NAFLD: Nonalcoholic fatty liver diseases
NF-Kb: Nuclear factor kappa B
Os: Oxidative stress
PA: Palmitate acid
PI: Physical inactivity
PI3K: Phosphatidylinositol 3-kinase
PKA: Protein kinase A
p38-MAPK: p38 mitogen-activated protein kinase
RNS: Reactive nitrogen species
ROS: Reactive oxygen species
SO: Sarcopenic obesity
SOD: Superoxide dismutase
TG: Triglyceride
TNF-*α*: Tumor necrosis factor-*α*
T2DM: Type 2 diabetes mellitus
UPR: Unfolded protein response
UPS: Ubiquitin-proteasome system
US: Ultrasound
V/Q: Ventilation and perfusion
WHO: World Health Organization
WHR: Waist-to-hip ratio
WHTR: Waist-to-height ratio.
